# Breast Cancer Histopathological Images Recognition Based on Low Dimensional Three-Channel Features

**DOI:** 10.3389/fonc.2021.657560

**Published:** 2021-06-14

**Authors:** Yan Hao, Shichang Qiao, Li Zhang, Ting Xu, Yanping Bai, Hongping Hu, Wendong Zhang, Guojun Zhang

**Affiliations:** ^1^ School of Information and Communication Engineering, North University of China, Taiyuan, China; ^2^ Department of Mathematics, School of Science, North University of China, Taiyuan, China; ^3^ School of Instrument and Electronics, Key Laboratory of Dynamic Testing Technology, North University of China, Taiyuan, China

**Keywords:** breast cancer, histopathological images recognition, feature extraction, low dimensional features, three-channel features

## Abstract

Breast cancer (BC) is the primary threat to women’s health, and early diagnosis of breast cancer is imperative. Although there are many ways to diagnose breast cancer, the gold standard is still pathological examination. In this paper, a low dimensional three-channel features based breast cancer histopathological images recognition method is proposed to achieve fast and accurate breast cancer benign and malignant recognition. Three-channel features of 10 descriptors were extracted, which are gray level co-occurrence matrix on one direction (GLCM1), gray level co-occurrence matrix on four directions (GLCM4), average pixel value of each channel (APVEC), Hu invariant moment (HIM), wavelet features, Tamura, completed local binary pattern (CLBP), local binary pattern (LBP), Gabor, histogram of oriented gradient (Hog), respectively. Then support vector machine (SVM) was used to assess their performance. Experiments on BreaKHis dataset show that GLCM1, GLCM4 and APVEC achieved the recognition accuracy of 90.2%-94.97% at the image level and 89.18%-94.24% at the patient level, which is better than many state-of-the-art methods, including many deep learning frameworks. The experimental results show that the breast cancer recognition based on high dimensional features will increase the recognition time, but the recognition accuracy is not greatly improved. Three-channel features will enhance the recognizability of the image, so as to achieve higher recognition accuracy than gray-level features.

## Introduction

Cancer has become one of the major public health problems that seriously threaten the health of people. The incidence and mortality of breast cancer have been rising continuously in recent years. Early accurate diagnosis is the key to improve the survival rate of patients. Mammogram is the first step of early diagnosis, but it is difficult to detect cancer in the dense breast of adolescent women, and the X-ray radiation poses a threat to the health of patients and radiologists. Computed tomography (CT) is a localized examination, which can not be used to judge that a patient is suffering from breast cancer according to the observed abnormalities. The gold standard for breast cancer diagnosis is still pathological examination. Pathological examinations usually obtain tumor specimens through puncture, excision, etc. And then stain them with hematoxylin and eosin (H&E) stains. Hematoxylin binds deoxyribonucleic acid (DNA) to highlight the nucleus, while eosin binds proteins and highlights other structures. Accurate diagnosis of breast cancer requires experienced histopathologists, and it requires a lot of time and effort to complete this task. In addition, the diagnosis results of different histopathologists are not the same, which strongly depends on the prior knowledge of histopathologists. It resulting in lower diagnosis consistency, and the average diagnosis accuracy is only 75% (1).

Currently, breast cancer diagnosis based on histopathological images is facing three major challenges. Firstly, there is a shortage of experienced histopathologists around the world, especially in some underdeveloped areas and small hospitals. Secondly, the diagnosis of histopathologist is subjective and there is no objective evaluation basis. Whether the diagnosis is correct or not depends entirely on the histopathologists’ prior knowledge. Thirdly, the diagnosis of breast cancer based on histopathological images is very complicated, time-consuming and labor-intensive, which is inefficient in the era of big data. In face of these problems, an efficient and objective breast cancer diagnosis method is urgently needed to alleviate the workload of histopathologists.

With the rapid development of computer-aided diagnosis (CAD), it has been gradually applied to the clinical field. The CAD system cannot completely replace the doctor, but it can be used as a “second reader” to assist doctors in diagnosing diseases. However, there are many false positive areas detected by the computer, which will take a lot of time of doctors to re-evaluate the results prompted by the computer, resulting in a decrease in the accuracy and efficiency. Therefore, how to improve the sensitivity of computer-aided tumor detection method, while greatly reducing the false positive detection rate, improve the overall performance of the detection method is a subject to be studied.

In recent years, machine learning has been successfully applied to image recognition, object recognition, and text classification. With the advancement of computer-aided diagnosis technology, machine learning has also been successfully applied to breast cancer diagnosis (2–8). There are two common methods, histopathological images classification based on artificial feature extraction and traditional machine learning methods, and histopathological images classification based on deep learning methods. Histopathological images classification based on artificial feature extraction and traditional machine learning methods needs manual design of features, but it does not require equipment with high performance and has advantages in computing time. However, histopathological images classification based on deep learning, especially convolutional neural network (CNN), often requires a large number of labeled training samples, while the labeled data is difficult to obtain. The labeling of lesions is a time-consuming and laborious work, which takes a lot of time even for very experienced histopathologists.

The key of traditional histopathological images classification is feature extraction. The common features include color features, morphological features, texture features, statistical features etc. Spanhol et al. (9) introduced a publicly available breast cancer histopathology dataset (BreaKHis), and they extracted LBP, CLBP, gray level co-occurrence matrix (GLCM), Local phase quantization (LPQ), parameter-free threshold adjacency statistics (PFTAS) and one keypoint descriptor named ORB features, and 1-nearest neighbor (1-NN), quadratic linear analysis (QDA), support vector machines (SVMs), and random forests (RF) were used to assess the aforementioned features, with an accuracy range from 80% to 85%. Pendar et al. (10) introduced a representation learning-based unsupervised domain adaptation on the basis of (9) and compared it with the results of CNN. Anuranjeeta et al. (11) proposed a breast cancer recognition method based on morphological features. 16 morphological features were extracted, and 8 classifiers were used for recognition, the accuracy is about 80%. The authors in (12–14) proposed breast cancer recognition methods based on texture features. Particularly, Carvalho et al. (14) used phylogenetic diversity indexes to characterize the types of breast cancer. Sudharshan et al. (15) compared 12 multi-instance learning methods based on PFTAS and verified that multi-instance learning is more effective than single-instance learning. But none of them considered the color channel of the image. Fang et al. (16) proposed a framework called Local Receptive Field based Extreme Learning Machine with Three Channels (3C-LRF-ELM), which can automatically extract histopathological features to diagnose whether there is inflammation. In addition, in order to reduce the recognition time and the complexity of the algorithms, this paper is committed to achieving high recognition accuracy with low dimensional features.

Deep learning methods, especially CNN, can achieve more accurate cancer recognition (17–25) for it’s ability to extract powerful high-level features compared with traditional image recognition methods. For example, Spanhol et al. (17) used the existing AlexNet to test the BreaKHis dataset, and its recognition accuracy was significantly higher than their previous work (9). The authors in (18–21, 25) used different CNN frameworks and obtained the recognition accuracy of more than 90% on the two-class problem of the BreaKHis dataset. Benhammou et al. (22) comprehensively surveyed the researches based on BreaKHis datasets from the magnification-specific binary, magnification independent binary, magnification specific multi-category and magnification independent multi-category four aspects, and proposed a magnification independent multi-category method based on CNN, which is rarely considered in previous studies. The works (23–26) also achieved good performance on the Bioimaging 2015 dataset. Both the BreaKHis and Bioimaging 2015 are the challenging datases for breast cancer detection. Due to the drawbacks of model training, most researchers’ research were based on models that have been well trained through other datasets and verified by histopathological images. Few people trained a complete model with histopathological images for the lack of labeled data.

**Figure 1:**
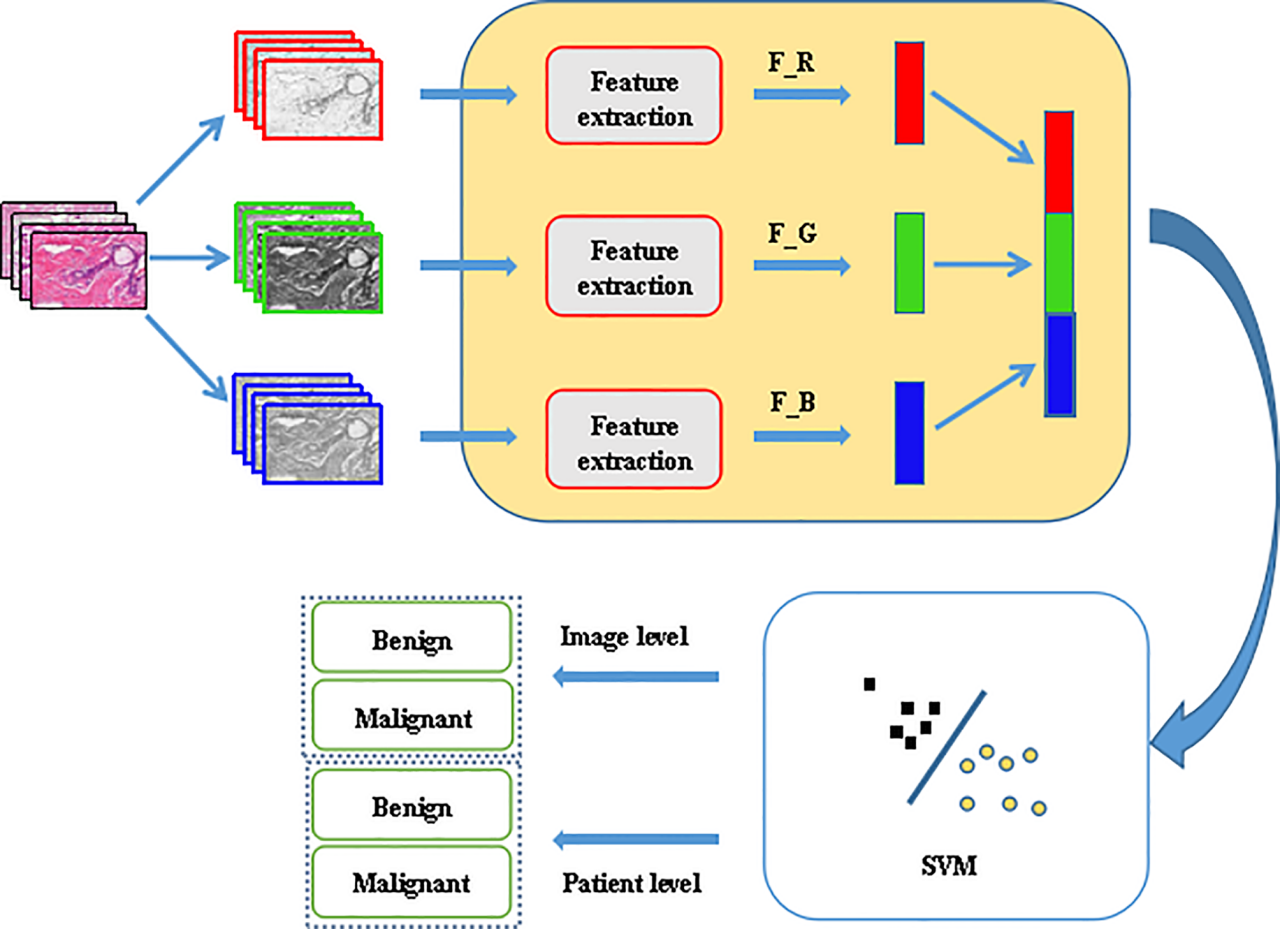
Proposed framework for histopathological image classification.

In order to reduce the workload of histopathologists and allow them to spend more time on the diagnosis of more complex diseases, efficient and fast computer-aided diagnosis methods are of urgent need. This paper proposed a breast cancer histopathological images recognition method based on low dimensional three-channel features. The features of the three channels of the image were extracted respectively, then the three-channel features were fused to realize better breast cancer histopathological images recognition for the image level and the patient level. The framework is shown in **Figure 1**.

The contributions of this paper are as follows:

1) proposed a histopathological images recognition method based on three-channel features,
2) proposed a histopathological images recognition method based on low dimensional features,
3) it is a method with high accuracy and fast recognition speed,
4) it is a method easy to implement.
The rest of the paper is organized as follows: in Section 2 the feature extraction methods are introduced, the experiments and results analysis are given in Section 3, and Section 4 concludes the work.

## Feature Extraction

### Gray Level Co-Occurrence Matrix

Gray level co-occurrence matrix is a common method to describe the texture of an image by studying its spatial correlation characteristics. In 1973, Haralick et al. first used GLCM to describe texture features (27). In our experiments, we calculated the GLCM of 256 gray levels in one direction 0° and four directions 0°, 45°, 90°, 135°, respectively. Then, according to the GLCM, 22 related features were calculated: autocorrelation, contrast, 2 correlation, cluster probability, cluster shade, dissimilarity, energy, entropy, 2 homogeneity, maximum probability, sum of squares, sum average, sum variance, sum entropy, difference variance, difference entropy, 2 information measures of correlation, inverse difference, inverse difference moment (27–29).

### Average Pixel Value of Each Channel

The average value reflects the centralized tendency of the data and is an important amplitude feature of images. For an image, the average pixel value of each color channel is expressed as

(1) $$f_{mean}=\frac{1}{MN}\sum_{x_{c}=1}^{M}\sum_{y_{c}=1}^{N}f(x_{c},y_{c}),$$

where *f* (*x_c_*, *y_c_* ) represents the pixel value of (*x_c_*, *y_c_* ).

### Hu Invariant Moment

Geometric moments were proposed by Hu.M.K (30) in 1962. They constructed seven invariant moments according to second-order and third-order normalized central moments, and proved that they are invariant to rotation, scaling and translation. Hu invariant moment is a region-based image shape descriptor. In the construction of Hu invariant moments, the central moment is used to eliminate the influence of image translation, the normalization eliminates the influence of image scaling, and the polynomial is constructed to realize the invariant characteristics of rotation. Different order moments reflect different characteristics, the low order reflects the basic shape of the target, and the high order reflects the details and complexity.

### Wavelet Features

The result of two-dimensional wavelet decomposition reflects the frequency changes in different directions and the texture characteristics of the image. Since the detail subgraph is the high-frequency component of the original image and contains the main texture information, the energy of the individual detail subgraph is taken as the texture feature, which reflects the energy distribution along the frequency axis with respect to the scale and direction. In this paper, 5-layer wavelet decomposition was carried out, and the energy of high-frequency components in each layer was taken as the feature vector.

### Tamura

Tamura et al. (31) proposed a texture feature description method based on the psychological research of texture visual perception, and defined six characteristics to describe texture. Namely, coarseness, contrast, directionality, line likeness, regu larity, and roughness. Coarseness reflects the change intensity of image gray level. The larger the texture granularity is, the coarser the texture image is. Contrast reflects the lightest and darkest gray levels in a gray image, and the range of differences determines the contrast. Directionality reflects the intensity of image texture concentration along a certain direction. Lineality reflects whether the image texture has a linear structure. Regulation reflects the consistency of texture features between a local region and the whole image. Roughness is the sum of roughness and contrast.

### Local Binary Pattern

Local Binary Pattern (32) is an operator used to describe local texture features of an image. It has significant advantages such as rotation invariance and gray level invariance. The original LBP operator is defined as comparing the gray values of eight adjacent pixels with the threshold value namely the center pixel in a 3×3 window. If the value of the adjacent pixel is greater than or equal to the value of the center pixel, the position of the pixel is marked as 1, otherwise it is 0. That is, for a pixel (*x_c_, y_c_*) on the image

(2) $$LBP_{P,R}\;(x_{c},y_{c})=\sum_{p=0}^{P-1}s(g_{p}-g_{c})2^{p},s(x)=\left\{ \begin{array}{l} 1,x\geq 0\\ 0,x<0 \end{array}\right.$$

Where *P* is the number of sampling points in the neighborhood of the center pixel, *R* is the radius of the neighborhood, *g_c_* is the gray value of the center pixel; *g_p_* is the gray value of the pixel adjacent to the center pixel.

In this way, 8 points in the neighborhood can be compared to generate a total of 256 8-bit binary numbers, that is, the LBP value of the center pixel of the 3×3 window is obtained, and this value is used to reflect the texture information of the region.

### Completed Local Binary Pattern

Completed local binary pattern (33) is a variant of LBP. The local area of the CLBP operator is represented by its center pixel and local differential sign magnitude transformation. After the center pixel is globally thresholded, it is coded with a binary string as CLBP_Center (CLBP_C). At the same time, the local difference sign magnitude transformation is decomposed into two complementary structural components: difference sign CLBP-Sign (CLBP_S) and difference magnitude CLBP-Magnitude (CLBP_M). For a certain pixel (*x_c_, y_c_*) on the image, the components are expressed as:

(3) $$\left\{ \begin{array}{l} CLBP\_C_{P,R}(x_{c},y_{c})=s(g_{c}-g_{N})\\ CLBP\_S_{P,R}(x_{c},y_{c})=\sum_{p=0}^{P-1}s(g_{p}-g_{c})2^{p}\;s(x)=\left\{ \begin{array}{l} 1,x\geq 0\\ 0,x<0 \end{array}\right.\\ CLBP\_M_{P,R}\left(x_{c},y_{c}\right)=\sum_{p=0}^{P-1}s(D_{p}-D_{c})2^{p} \end{array}\right..$$

Where, *N* is the number of windows, $g_{N}=\frac{1}{N}\sum_{n=0}^{N-1}g_{n}$represents the mean gray value about *g_c_* when the center point is constantly moving, and $D_{p}=\left|g_{p}-g_{c}\right|$,$D_{c}=\frac{1}{P}\sum_{p=0}^{P-1}\left|g_{p}-g_{c}\right|$represents the mean magnitude. *CLBP_S_P,R_* (*x_c_, y_c_*) is equivalent to the traditional LBP operator, which describes the difference sign characteristics of the local window. *CLBP_M_P,R_* (*x_c_, y_c_*) describes the difference magnitude characteristics of the local window. *CLBP_C_P,R_* (*x_c_, y_c_*) is the gray level information reflected by the pixel at the center. In our experiments, we worked with rotation-invariant uniform patterns, with a standard value of *P* = 8, *R* = 1, yielding a 20-D feature vector for each channel.

### Gabor

Gabor feature is a kind of feature that can be used to describe the texture information of image. The frequency and direction of Gabor filter are similar to human visual system, and it is particularly suitable for texture representation and discrimination. Gabor features mainly rely on Gabor kernel to window the signal in frequency domain, so as to describe the local frequency information of the signal. Different textures generally have different center frequencies and bandwidths. According to these frequencies and bandwidths, a set of Gabor filters can be designed to filter texture images. Each Gabor filter only allows the texture corresponding to its frequency to pass smoothly, while the energy of other textures is suppressed. Texture features are analyzed and extracted from the output results of each filter for subsequent classification tasks. we used the Gabor filters with five scales and eight orientations, the size of the filter bank is 39×39, the block size is 46×70, yielding a 4000-D feature vector for each channel.

### Histogram of Oriented Gradient

Histogram of Oriented Gradient (34) is a feature descriptor used for object detection in computer vision and image processing. It constructs features by calculating and counting the histogram of the gradient direction in the local area of the image. The use of gradient information can well reflect the edge information of the target, the local appearance and shape of the image can be characterized by the size of the local gradient. It is generally used in pedestrian detection, face recognition and other fields, but it does not perform well on images with complex texture information. It is introduced as a comparison in this paper.

## Experiments and Results

### Dataset

**Figure 2:**
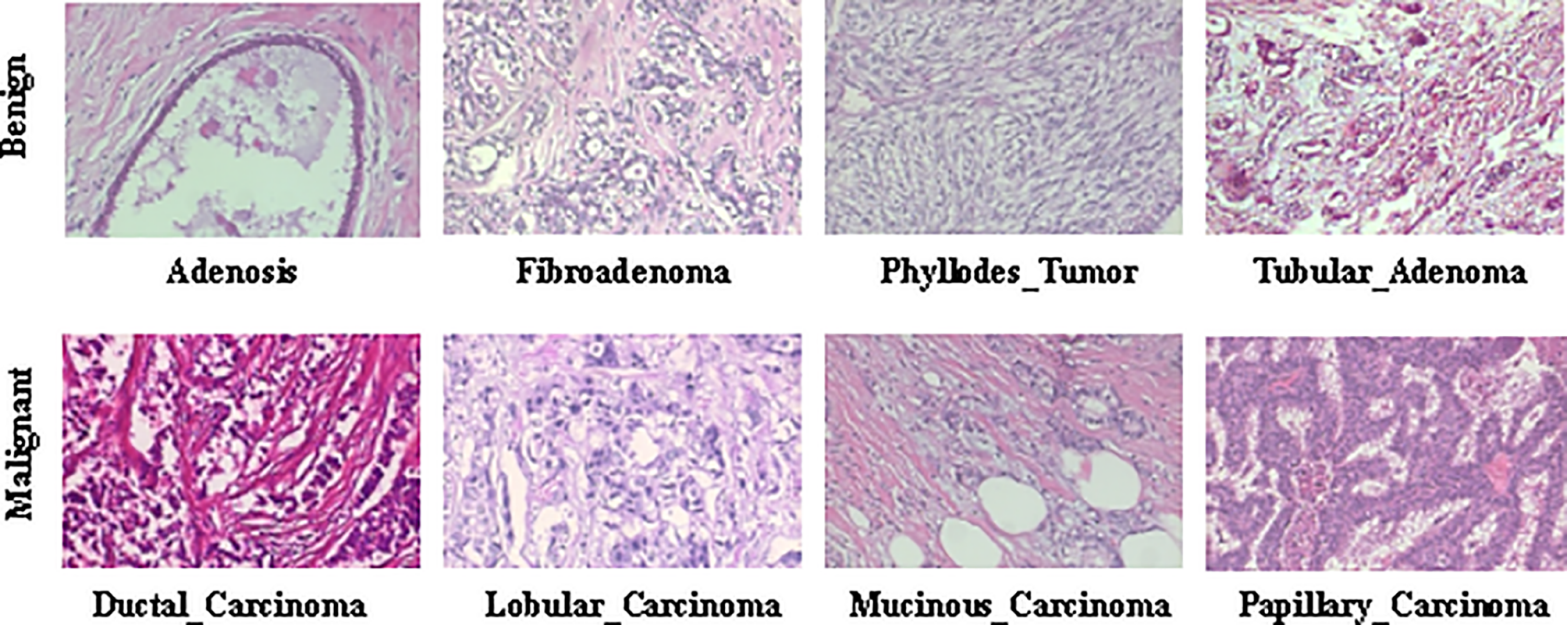
Representative examples of BreaKHis dataset.

The BreaKHis dataset (9) contains biopsy images of benign and malignant breast tumors, which were collected through clinical studies from January 2014 to December 2014. During the period, all patients with clinical symptoms of BC were invited to the Brazilian P&D laboratory to participate in the study. Samples were collected by surgical open biopsy (SOB) and stained with hematoxylin and eosin. Hematoxylin is alkaline, mainly making the chromatin in the nucleus and nucleic acid in the cytoplasm stained blue-purple. eosin is acidic, mainly making the components in the cytoplasm and extracellular matrix stained pink. These images can be used for histological studies and marked by pathologists in the P&D laboratory. The BreaKHis dataset consists of 7909 breast tumor tissue microscopic images of 82 patients, divided into benign and malignant tumors, including 2480 benign (24 patients) and 5429 malignant (58 patients). The image is obtained in a three-channel RGB (red-green-blue) true color space with magnification factors of 40X, 100X, 200X, 400X, and the size of each image is 700×460. **Tables 1** and **2** summarize the image distribution. And **Figure 2** shows the representative examples of BreaKHis dataset.

**Table 1:** Image distribution by magnification factor and class.

Magnification	Benign	Malignant	Total
40X	625	1370	1995
100X	644	1437	2081
200X	623	1390	2013
400X	588	1232	1820
Total	2480	5429	7909
Patients	24	58	82

**Table 2:** Image distribution by magnification factor and subclass.

Class	Sub-class	Magnification			
		40X	100X	200X	400X
Benign	Adenosis (A)	114	113	111	106
	Fibroadenoma(F)	253	260	264	237
	Phyllodes_tumor(PT)	109	121	108	115
	Tubular_adenoma(TA)	149	150	140	130
Malignant	Ductal_carcinoma(DC)	864	903	896	788
	Lobular_carcinoma(LC)	156	170	163	137
	Mucinous_carcinoma(MC)	205	222	196	169
	Papillary_carcinoma(PC)	145	142	135	138

### Protocol

All of the experiments were conducted on a platform with an Intel Core i7-5820K CPU and 16G memory. The BreaKHis dataset has been randomly divided into a training set (70%, 56 patients) and a testing set (30%, 26 patients). We guarantee that patients use to build the training set are not used for the testing set. The results presented in this work are the average of five trials.

All the images we used were without any preprocessing before feature extraction. For the SVM, we chose the RBF kernel. The best penalty factor *c*=2 and kernel function parameter *g*=1 were obtained by cross validation. For wavelet function, we selected coif5 wavelet function, which has better symmetry than dbN, has the same support length as db3N and sym3N, and has the same number of vanishing moments as db2N and sym2N.

Here, we report the recognition accuracy at both the image level and the patient level. For the image level, let *N*_rec_I_ be the number of images correctly classified, *N* represents all the test samples, then the recognition accuracy of the image level can be defined as

(4) $$Image\_accuracy=\frac{N_{rec\_I}}{N}.$$

For the patient level, we followed the definition of (9). Let *N_P_* be the image of patient P, *S* is the total number of patients, and *N_rec_P_* images of patient P were correctly classified, then the patient score can be defined as

(5) $$Patient\;score=\frac{N_{rec\_P}}{N_{P}},$$

and define the recognition accuracy of the patient level as

(6) $$Patient\_accuracy=\frac{\sum Patient\;score}{S}.$$

To further assess the performance of the proposed framework, sensitivity (Se), precision (Pr) and F1-score metrics were used and the formulations of the metrics are described as

(7) $$Se=\frac{TP}{TP+FN},$$

(8) $$Pr=\frac{TP}{TP+FP},$$

(9) $$F1-score=\frac{2\times TP}{2\times TP+FP+FN},$$

where true positive (TP) represents the number of malignant samples classified as malignant, whereas true negative (TN) represents the number of benign samples classified as benign. Also, false positive (FP) represents the number of benign samples incorrectly classified as malignant while false negative (FN) represents the number of malignant samples misclassified as benign.

### Experiment Results

**Table 3** reports the performance of all descriptors we have assessed. The image level recognition accuracy, the patient level recognition accuracy, sensitivity, precision and F1-score of 10 different three-channel descriptors under 4 magnifications were compared. The descriptors are GLCM1, GLCM4, APVEC, HIM, wavelet feature, Tamura, CLBP. In order to show the effectiveness of low dimensional features, LBP, Gabor, and Hog were introduced for comparison.

**Table 3:** Classification performance of different descriptors based on three-channel features.

Features	Magnification	Image_accuracy (%)	Patient_accuracy (%)	Sensitivity (%)	Precision (%)	F1-score (%)
GLCM1	40X	94.12 ± 2.19	93.48 ± 2.70	94.99 ± 1.53	**97.01 ± 2.31**	95.98 ± 1.73
	100X	92.65 ± 3.08	91.74 ± 3.89	95.61 ± 4.81	94.53 ± 1.56	95.01 ± 2.47
	200X	94.67 ± 2.02	**94.24 ± 2.86**	97.17 ± 3.49	95.81 ± 2.01	96.44 ± 1.54
	400X	90.98 ± 2.17	91.43 ± 2.19	91.80 ± 4.09	95.76 ± 3.01	93.66 ± 1.81
GLCM4	40X	93.42 ± 3.54	92.95 ± 4.02	94.72 ± 3.94	96.26 ± 2.22	95.46 ± 2.83
	100X	91.98 ± 3.79	91.16 ± 3.88	96.57 ± 3.62	92.85 ± 2.76	94.65 ± 2.86
	200X	93.53 ± 3.50	92.81 ± 4.72	96.14 ± 3.98	95.36 ± 2.75	95.70 ± 2.37
	400X	90.65 ± 2.93	90.76 ± 3.28	93.54 ± 3.49	93.72 ± 3.76	93.56 ± 2.20
APVEC	40X	92.12 ± 1.09	90.55 ± 0.84	94.67 ± 2.04	94.83 ± 1.31	94.72 ± 0.66
	100X	90.20 ± 2.33	89.18 ± 3.45	94.05 ± 2.36	93.16 ± 4.15	93.52 ± 1.40
	200X	**94.97 ± 1.35**	94.21 ± 2.37	**98.30 ± 0.73**	95.21 ± 1.79	**96.72 ± 0.84**
	400X	92.78 ± 3.14	93.30 ± 3.25	94.07 ± 4.91	96.04 ± 2.54	94.96 ± 2.35
HIM	40X	89.21 ± 1.59	86.44 ± 3.46	93.96 ± 2.36	91.81 ± 2.46	92.85 ± 2.31
	100X	88.99 ± 2.45	87.67 ± 3.14	94.17 ± 3.38	91.62 ± 3.84	92.78 ± 1.48
	200X	92.93 ± 1.89	92.19 ± 2.87	95.06 ± 2.28	95.59 ± 1.60	95.30 ± 1.20
	400X	88.64 ± 3.97	88.61 ± 4.94	91.77 ± 6.21	92.73 ± 2.11	92.14 ± 3.03
Wavelet	40X	80.98 ± 4.23	80.03 ± 7.16	97.66 ± 4.23	80.78 ± 8.00	88.38 ± 4.81
	100X	80.36 ± 3.66	80.24 ± 1.03	97.02 ± 4.62	80.82 ± 4.39	88.04 ± 2.53
	200X	78.99 ± 4.47	76.50 ± 3.33	97.37 ± 3.63	79.46 ± 4.22	87.43 ± 2.86
	400X	76.08 ± 2.22	76.79 ± 1.45	89.65 ± 4.31	80.27 ± 3.99	84.56 ± 1.74
Tamura	40X	78.91 ± 3.30	78.62 ± 1.27	97.23 ± 3.93	79.30 ± 6.16	87.31 ± 2.17
	100X	78.68 ± 4.03	78.09 ± 1.17	99.27 ± 0.43	78.18 ± 4.08	87.43 ± 2.66
	200X	77.37 ± 1.89	76.00 ± 2.09	94.43 ± 3.02	79.41 ± 2.38	86.23 ± 1.59
	400X	75.88 ± 2.86	75.66 ± 1.72	94.04 ± 2.18	77.68 ± 2.64	85.06 ± 2.18
LBP	40X	84.38 ± 2.32	86.51 ± 2.43	93.23 ± 2.92	87.07 ± 3.48	89.87 ± 2.37
	100X	83.91 ± 4.84	85.20 ± 3.78	95.95 ± 2.63	84.66 ± 5.15	89.89 ± 3.33
	200X	83.26 ± 4.04	84.05 ± 3.27	92.24 ± 4.23	86.39 ± 4.03	89.15 ± 3.13
	400X	82.35 ± 5.56	82.76 ± 4.84	91.64 ± 4.12	85.57 ± 6.45	88.35 ± 3.81
CLBP	40X	82.63 ± 3.54	83.18 ± 3.68	93.29 ± 3.77	85.03 ± 6.84	88.89 ± 2.95
	100X	82.64 ± 4.69	84.31 ± 3.66	95.46 ± 3.93	83.83 ± 5.31	89.14 ± 3.10
	200X	78.72 ± 2.61	78.20 ± 2.36	95.65 ± 4.81	80.13 ± 3.58	87.08 ± 1.91
	400X	75.26 ± 4.01	75.97 ± 2.28	94.39 ± 5.78	77.40 ± 5.36	84.81 ± 2.42
Gabor	40X	86.11 ± 4.46	84.87 ± 4.85	97.00 ± 1.34	86.45 ± 6.92	91.29 ± 3.20
	100X	89.98 ± 2.15	89.79 ± 2.74	93.45 ± 2.87	93.37 ± 3.78	93.33 ± 1.39
	200X	91.04 ± 2.66	89.65 ± 3.97	97.14 ± 1.86	91.58 ± 3.78	94.23 ± 1.72
	400X	88.94 ± 2.87	87.84 ± 2.69	96.96 ± 1.74	88.94 ± 3.07	92.75 ± 1.99
Hog	40X	76.59 ± 4.42	76.82 ± 7.21	95.42 ± 2.97	78.68 ± 10.21	85.94 ± 4.74
	100X	76.06 ± 4.00	75.54 ± 2.64	95.13 ± 5.36	78.21 ± 5.80	85.59 ± 2.50
	200X	76.83 ± 3.35	76.28 ± 2.18	93.32 ± 3.38	79.60 ± 4.38	85.81 ± 2.30
	400X	77.88 ± 3.23	78.20 ± 1.72	89.69 ± 4.25	82.28 ± 5.96	85.59 ± 1.94

The bold values indicate the best value of each metric.

For images at 40X magnification, GLCM1 achieved the highest recognition accuracy of 94.12 ± 2.19% at the image level and 93.48 ± 2.7% at the patient level, as well as the highest precision and F1_score. The second was GLCM4 with which the image_accuracy and the patient_accuracy were 93.4 ± 3.54% and 92.95 ± 4.02, respectively. Followed by APVEC achieving the image_accuracy of 92.12 ± 1.09%, and the patient_accuracy of 90.55 ± 0.84%. The same conclusion was drawn for 100X. The image level recognition accuracy and the patient level recognition accuracy of GLCM1, GLCM4, and APVEC were 92.65 ± 3.08%, 91.74 ± 3.89%, 91.98 ± 3.79%, 91.16 ± 3.88%, 90.2 ± 2.33%, 89.18 ± 3.45%, respectively. However, for 200X, APVEC achieved the highest image level recognition accuracy of 94.97 ± 1.35%, followed by GLCM1 and GLCM4. GLCM1 performed best at the patient level with an accuracy of 94.24 ± 2.86%, which is 0.3% higher than APVEC. As for 400X, APVEC performed best at both the image level (92.78 ± 3.14%) and the patient level (93.3 ± 3.25%) followed by GLCM1 and GLCM4. On the whole, GLCM1, GLCM4 and APVEC performed well at both the image level and the patient level, followed by HIM. The four descriptors all get the highest recognition accuracy at 200X, and all descriptors except Gabor and Hog obtain the worst performance at 400X, which is same as the conclusion of (18, 35). Although the recognition accuracy of LBP and Gabor is above 82%, which is also acceptable, it also needs more recognition time due to the high feature dimension, as shown in **Table 4**. Tamura and Hog performed slightly worse compared to other descriptors.

**Table 4:** Running time for feature extraction of each image and classification of different descriptors.

Methods	Feature dimensions	Running time for feature extraction of each image (s)	Running time for classification (s)
GLCM1	22×3-D	0.26	0.30
GLCM4	88×3-D	0.05	1.34
APVEC	1×3-D	10.29	0.05
HIM	7×3-D	0.19	0.12
Wavelet	5×3-D	0.41	0.10
Tamura	6×3-D	59.17	0.55
CLBP	20×3-D	0.61	0.19
LBP	256×3-D	0.47	3.09
Gabor	4000×3-D	12.48	66.80
Hog	288×3-D	0.21	4.10

**Figure 3:**
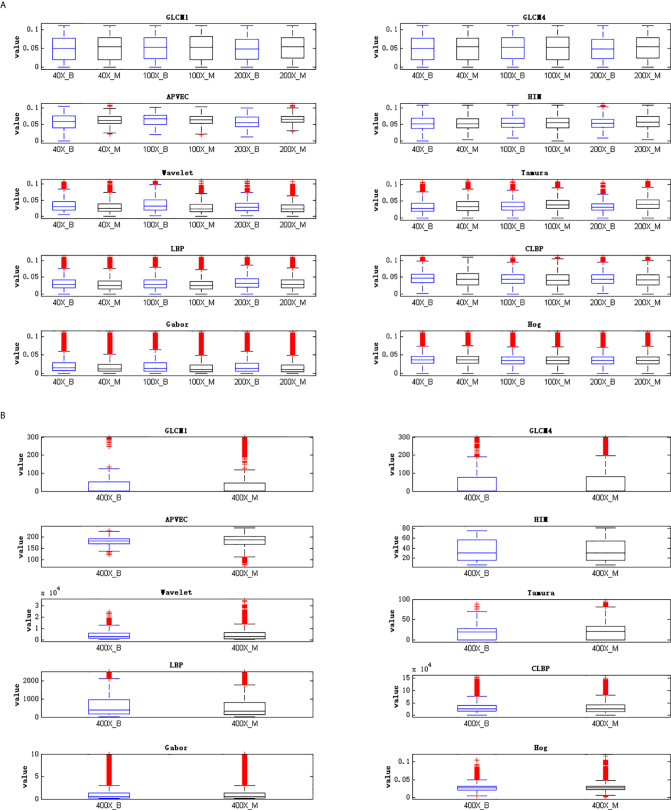
Visualization of feature distribution. **(A)** Feature distribution of 40X, 100X, 200X, **(B)** feature distribution of 400X.

The reason for the above results is that the distributions of features extracted by different feature descriptors are different. The high dispersion of feature distribution will increase the difficulty of image recognition, and the feature with more concentrated distribution will achieve better recognition performance. **Figure 3** is the best illustration of the results.

**Figure 3** is the visualization of feature distribution. The ordinate represents the feature values. Since the feature values of 40X, 100X, and 200X are relatively small, while the feature values of 400X are relatively large, the feature distribution cannot be displayed in the same figure at the same time. Here are two figures showing the data distribution, **Figure 3**(A) shows the feature distribution of 40X, 100X, 200X, and **Figure 3**(B) shows the feature distribution of 400X. It can be seen from **Figure 3** that for 40X, 100X, 200X, the outliers of GLCM1, GLCM4, APVEC, and HIM are much less than other feature descriptors, indicating that the distributions of these four features are relatively concentrated, which is beneficial for breast cancer identification. In addition, comparing the feature distributions of benign and malignant samples under different magnifications, it can be found that the data distribution of benign and malignant samples of Hog are very similar, indicating the weak ability to discriminate between benign and malignant, which is also the reason for its poor performance. The outliers of GLCM1 and GLCM4 under 400X are obviously more compared to other magnifications, and the similarity of the benign and malignant feature distributions of all descriptors is relatively high, resulting in the poor performance of 400X.

Compared with RGB images, grayscale images only retain the brightness information of the images, but lose the chroma and saturation information of the images. Three-channel features can make up for the lost information of single-channel features, increasing the recognition capability of features, so as to achieve better recognition performance. To further illustrate the advantages of three-channel features, **Table 5** shows the performance of different descriptors of gray-level features.

**Table 5:** Classification performance of different gray-level features.

Features	Magnification	Image_accuracy (%)	Patient_accuracy (%)	Sensitivity (%)	Precision (%)	F1-score (%)
GLCM1	40X	82.88 ± 5.27	83.48 ± 3.65	97.39 ± 4.75	83.08 ± 6.41	89.47 ± 2.98
	100X	83.71 ± 4.66	85.07 ± 2.69	99.45 ± 0.69	82.53 ± 4.93	90.12 ± 2.87
	200X	77.56 ± 4.64	76.44 ± 4.02	99.31 ± 0.83	77.44 ± 4.35	86.95 ± 2.54
	400X	78.51 ± 6.32	80.02 ± 4.46	96.45 ± 2.45	79.42 ± 5.33	86.86 ± 3.22
GLCM4	40X	82.06 ± 4.14	82.71 ± 4.75	98.61 ± 0.94	81.48 ± 3.75	89.14 ± 2.20
	100X	82.78 ± 4.69	83.58 ± 3.50	99.66 ± 1.17	81.56 ± 4.02	89.64 ± 2.42
	200X	77.38 ± 5.02	76.35 ± 3.46	99.18 ± 1.12	77.38 ± 3.95	86.85 ± 2.30
	400X	80.93 ± 6.43	82.37 ± 6.44	93.09 ± 3.70	83.28 ± 4.88	87.75 ± 3.46
APVEC	40X	74.83 ± 3.31	73.06 ± 4.12	99.86 ± 0.30	74.88 ± 5.58	85.56 ± 3.51
	100X	75.41 ± 3.78	73.73 ± 3.22	99.5 ± 0.66	75.51 ± 5.37	85.81 ± 3.33
	200X	75.39 ± 3.11	73.27 ± 0.49	99.86 ± 0.98	75.42 ± 3.51	85.91 ± 2.04
	400X	75.17 ± 3.62	75.58 ± 2.52	99.16 ± 1.43	75.06 ± 4.69	85.40 ± 2.60
HIM	40X	74.89 ± 3.27	73.17 ± 2.05	100.00 ± 1.83	74.88 ± 5.01	85.60 ± 2.54
	100X	76.06 ± 4.60	74.70 ± 2.91	99.06 ± 1.19	76.31 ± 5.51	86.11 ± 3.10
	200X	75.36 ± 3.08	73.27 ± 0.33	99.54 ± 0.94	75.51 ± 2.95	85.85 ± 2.03
	400X	73.97 ± 3.31	74.03 ± 1.41	99.48 ± 1.63	74.00 ± 4.42	84.83 ± 2.36
Wavelet	40X	81.47 ± 4.95	81.38 ± 1.56	99.41 ± 0.74	80.50 ± 3.77	88.90 ± 2.58
	100X	80.54 ± 3.78	80.51 ± 1.16	99.80 ± 0.16	79.50 ± 3.66	88.45 ± 2.27
	200X	77.50 ± 4.04	76.55 ± 0.73	99.09 ± 2.14	77.41 ± 4.10	86.87 ± 1.90
	400X	74.82 ± 3.87	74.77 ± 2.90	98.15 ± 1.54	75.15 ± 5.11	85.08 ± 2.90
Tamura	40X	78.55 ± 4.12	77.83 ± 1.05	99.08 ± 1.75	78.16 ± 3.94	87.33 ± 2.17
	100X	79.45 ± 4.07	79.16 ± 1.61	99.27 ± 1.28	78.85 ± 4.40	87.83 ± 2.41
	200X	76.54 ± 2.14	75.33 ± 2.56	98.60 ± 1.59	76.81 ± 2.50	86.33 ± 1.53
	400X	73.30 ± 2.98	73.08 ± 2.16	100.00 ± 0.64	73.30 ± 4.24	84.57 ± 2.58
LBP	40X	84.97 ± 3.54	87.04 ± 3.96	94.61 ± 4.40	86.51 ± 1.54	90.31 ± 2.79
	100X	84.31 ± 4.98	86.05 ± 3.24	97.31 ± 1.54	84.31 ± 4.31	90.25 ± 3.03
	200X	83.12 ± 4.99	84.17 ± 5.72	92.48 ± 4.47	86.04 ± 2.53	89.09 ± 3.39
	400X	80.05 ± 5.54	80.72 ± 4.94	91.65 ± 4.58	82.93 ± 4.95	86.99 ± 3.97
CLBP	40X	83.19 ± 5.15	84.63 ± 3.17	96.40 ± 1.73	83.89 ± 6.26	89.57 ± 3.07
	100X	84.06 ± 4.80	86.12 ± 2.23	97.77 ± 2.25	83.89 ± 6.08	90.17 ± 3.01
	200X	78.56 ± 3.55	78.58 ± 3.30	96.38 ± 3.92	79.75 ± 8.05	87.12 ± 3.13
	400X	74.79 ± 3.80	74.94 ± 4.33	97.74 ± 4.66	75.19 ± 8.13	84.98 ± 3.59
Gabor	40X	80.06 ± 4.64	79.84 ± 2.61	98.03 ± 2.59	79.96 ± 5.82	88.01 ± 2.55
	100X	81.25 ± 4.89	81.57 ± 2.57	98.91 ± 1.94	80.6 ± 5.84	88.75 ± 3.01
	200X	76.94 ± 2.88	75.43 ± 1.90	98.17 ± 1.71	77.36 ± 4.68	86.49 ± 2.23
	400X	77.40 ± 4.68	78.70 ± 3.54	92.37 ± 2.37	80.19 ± 7.64	85.71 ± 3.28
Hog	40X	76.46 ± 4.03	76.30 ± 2.29	97.50 ± 1.98	77.18 ± 4.00	86.09 ± 2.31
	100X	75.80 ± 3.51	74.31 ± 1.73	97.63 ± 2.91	76.56 ± 3.87	85.76 ± 2.27
	200X	76.22 ± 2.59	74.74 ± 1.60	97.78 ± 1.86	76.85 ± 2.43	86.05 ± 1.81
	400X	76.02 ± 3.23	75.84 ± 1.84	91.15 ± 4.68	79.30 ± 4.34	84.70 ± 2.76

**Figure 4:**
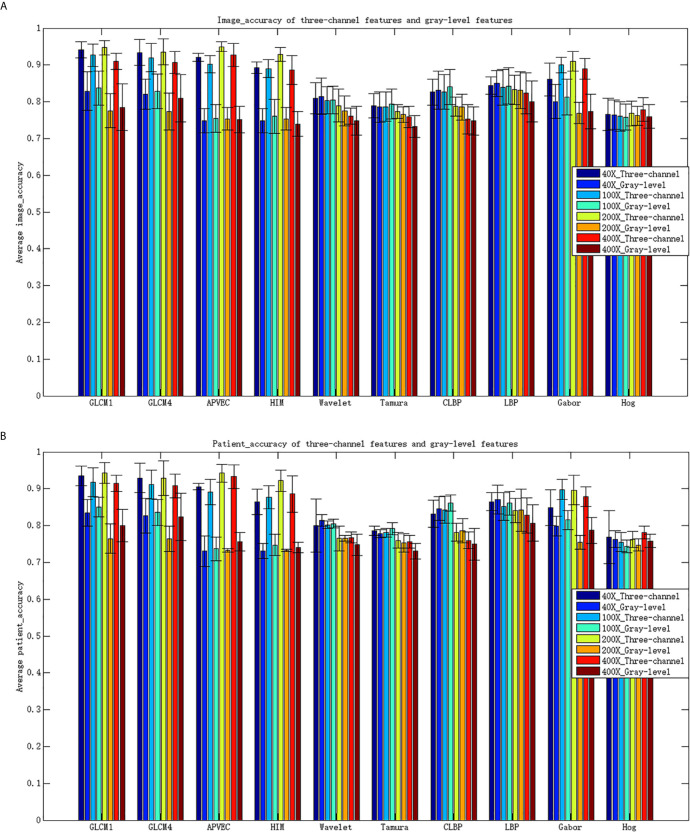
Classification accuracy for different features. **(A)** Image_accuracy for three-channel features and gray-level features, **(B)** patient_accuracy for three-channel features and gray-level feature\s.

Comparing **Table 3** and **Table 5**, it can be seen that the performance of the three-channel features is much better than that of gray-level features, especially GLCM1, GLCM4, APVEC, HIM and Gabor. The accuracy for most of them has increased by more than 10% for both the image level and the patient level. **Figure 4** shows the average recognition accuracy of three-channel features and gray-level features for the image level and the patient level. The advantages of the three-channel features can be seen more clearly from **Figure 4**.

Although the advantages of the three-channel features are obvious, we still have no idea about which channel plays a more important role in the classification results. **Table 6** shows the classification performance of single-channel features under different magnifications. Observing the experimental results, we can find that R channel have a greater impact on the classification results under 40X, 100X, 200X magnifications, while B channel performs better under 400X. This is consistent with the actual situation of H&E histopathological images under different magnifications. The images of 40X, 100X, and 200X have more cytoplasm and appear pink. The image of 400X contains more information about the precise lesion locations, which is usually presented through the nucleus, and appear blue-purple.

**Table 6:** Classification performance of single-channel features under different magnifications.

Features	40X					
	R		G		B	
	Image_accuracy (%)	Patient_accuracy (%)	Image_accuracy (%)	Patient_accuracy (%)	Image_accuracy (%)	Patient_accuracy (%)
GLCM1	89.03 ± 2.28	87.46 ± 2.23	82.25 ± 4.51	82.03 ± 2.30	83.62 ± 3.07	82.84 ± 2.12
GLCM4	88.47 ± 1.99	86.06 ± 1.58	81.53 ± 4.12	82.05 ± 2.68	83.58 ± 3.62	82.00 ± 3.23
APVEC	83.43 ± 1.51	79.73 ± 1.30	77.00 ± 3.95	77.39 ± 2.60	74.97 ± 3.32	73.18 ± 0.23
HIM	81.08 ± 1.03	77.26 ± 0.86	77.30 ± 4.28	77.81 ± 2.54	74.83 ± 3.31	73.08 ± 0.00
Wavelet	80.74 ± 4.69	80.24 ± 2.28	81.35 ± 5.17	81.21 ± 3.16	81.28 ± 5.12	80.97 ± 2.89
Tamura	76.90 ± 3.50	75.57 ± 0.64	79.32 ± 4.22	78.84 ± 1.85	77.17 ± 3.06	75.97 ± 0.77
LBP	84.70 ± 3.75	86.65 ± 3.63	84.11 ± 3.52	85.53 ± 2.93	84.64 ± 3.01	86.35 ± 2.02
CLBP	82.64 ± 4.17	83.29 ± 2.78	83.79 ± 3.75	84.90 ± 2.81	83.36 ± 4.40	84.18 ± 3.11
Gabor	83.46 ± 4.47	82.09 ± 3.56	78.63 ± 3.25	78.31 ± 3.32	80.60 ± 4.86	80.12 ± 2.49
Hog	76.59 ± 4.22	76.09 ± 3.13	76.38 ± 3.95	75.77 ± 1.91	76.07 ± 3.79	75.33 ± 2.18
**Features**	**100X**					
	**R**		**G**		**B**	
	**Image_accuracy(%)**	**Patient_accuracy(%)**	**Image_accuracy(%)**	**Patient_accuracy(%)**	**Image_accuracy(%)**	**Patient_accuracy(%)**
GLCM1	90.32 ± 1.59	88.52 ± 1.87	84.00 ± 4.99	84.95 ± 4.6	87.43 ± 1.10	85.00 ± 1.05
GLCM4	88.94 ± 1.46	86.83 ± 2.17	83.17 ± 4.99	84.62 ± 3.67	87.14 ± 1.54	85.58 ± 1.03
APVEC	77.70 ± 4.37	74.96 ± 2.26	78.29 ± 5.93	78.91 ± 4.77	75.00 ± 3.74	73.08 ± 0.00
HIM	78.83 ± 2.58	75.92 ± 1.32	78.32 ± 4.74	77.11 ± 3.99	76.17 ± 2.90	73.70 ± 0.44
Wavelet	79.94 ± 3.77	79.73 ± 1.38	81.00 ± 3.84	81.08 ± 1.01	79.46 ± 4.64	79.13 ± 2.12
Tamura	78.01 ± 3.83	77.31 ± 1.67	79.91 ± 4.28	79.69 ± 1.72	78.83 ± 4.26	78.41 ± 1.99
LBP	84.48 ± 4.98	85.68 ± 3.83	84.61 ± 5.02	86.51 ± 3.21	83.25 ± 4.49	84.54 ± 2.32
CLBP	81.75 ± 4.38	82.78 ± 3.12	81.41 ± 4.13	83.49 ± 1.73	81.83 ± 3.58	82.66 ± 1.55
Gabor	88.42 ± 2.99	87.27 ± 2.69	82.25 ± 5.07	83.63 ± 3.32	83.45 ± 3.64	83.31 ± 1.63
Hog	76.04 ± 3.86	74.73 ± 1.97	75.76 ± 4.05	74.40 ± 0.65	75.67 ± 3.93	74.34 ± 1.15
**Features**	**200X**					
	**R**		**G**		**B**	
	**Image_accuracy(%)**	**Patient_accuracy(%)**	**Image_accuracy(%)**	**Patient_accuracy(%)**	**Image_accuracy(%)**	**Patient_accuracy(%)**
GLCM1	91.31 ± 3.14	90.11 ± 3.82	79.05 ± 3.82	77.86 ± 2.81	88.62 ± 3.76	86.74 ± 5.37
GLCM4	90.45 ± 2.90	89.35 ± 3.65	78.18 ± 3.86	78.42 ± 3.27	88.77 ± 3.30	86.91 ± 4.72
APVEC	83.95 ± 0.97	81.71 ± 2.93	79.40 ± 5.22	79.00 ± 4.83	76.46 ± 1.57	73.89 ± 1.12
HIM	83.89 ± 1.56	81.72 ± 2.61	78.46 ± 5.16	77.63 ± 4.28	79.09 ± 2.46	77.02 ± 1.37
Wavelet	77.67 ± 4.12	76.68 ± 2.27	77.69 ± 3.76	76.58 ± 2.48	76.59 ± 3.99	75.16 ± 2.18
Tamura	75.98 ± 2.10	74.17 ± 2.44	76.48 ± 1.77	74.84 ± 2.52	76.01 ± 2.08	74.05 ± 2.18
LBP	83.21 ± 4.75	84.35 ± 3.95	82.66 ± 4.97	83.36 ± 4.19	82.48 ± 4.07	82.87 ± 3.39
CLBP	79.21 ± 3.43	79.24 ± 2.83	77.08 ± 3.15	75.97 ± 2.15	77.59 ± 3.37	77.19 ± 1.29
Gabor	88.38 ± 1.91	86.82 ± 2.72	78.46 ± 4.83	77.66 ± 3.66	84.03 ± 1.9	80.46 ± 2.54
Hog	75.75 ± 2.97	74.3 ± 1.25	76.39 ± 2.55	74.99 ± 1.12	75.99 ± 2.91	74.16 ± 1.06
**Features**	**400X**					
	**R**		**G**		**B**	
	**Image_accuracy(%)**	**Patient_accuracy(%)**	**Image_accuracy(%)**	**Patient_accuracy(%)**	**Image_accuracy(%)**	**Patient_accuracy(%)**
GLCM1	83.48 ± 1.11	81.67 ± 1.35	80.22 ± 5.84	81.96 ± 5.05	83.09 ± 2.20	82.28 ± 3.23
GLCM4	83.14 ± 1.11	81.24 ± 1.55	82.25 ± 5.36	83.43 ± 5.00	84.51 ± 2.73	83.75 ± 3.71
APVEC	73.34 ± 2.99	73.10 ± 0.05	79.31 ± 6.57	80.75 ± 5.89	73.37 ± 2.85	73.03 ± 0.11
HIM	74.27 ± 3.01	74.07 ± 1.10	78.08 ± 5.87	79.33 ± 5.53	76.01 ± 1.36	74.51 ± 2.39
Wavelet	75.47 ± 3.87	75.80 ± 2.41	75.18 ± 3.90	75.20 ± 2.50	75.54 ± 4.67	75.83 ± 3.40
Tamura	73.34 ± 2.99	73.10 ± 0.05	73.47 ± 2.76	73.25 ± 0.29	75.37 ± 4.01	75.92 ± 2.36
LBP	80.81 ± 5.01	81.87 ± 3.61	80.78 ± 5.77	82.31 ± 3.97	82.3 ± 6.32	83.35 ± 5.19
CLBP	74.88 ± 3.31	75.00 ± 1.70	74.07 ± 3.55	74.40 ± 1.21	74.63 ± 3.50	75.11 ± 1.60
Gabor	78.71 ± 3.73	76.82 ± 4.35	80.12 ± 5.34	81.46 ± 4.02	79.27 ± 2.88	77.86 ± 2.79
Hog	75.41 ± 3.02	74.66 ± 0.76	76.75 ± 3.68	76.91 ± 1.54	77.03 ± 3.31	76.62 ± 1.83

Different descriptors extract different features. It often cannot obtain all the effective information of the image only by one method. There may be a complementary relationship between different methods, and sometimes more redundant information may be added. In this paper, GLCM1 with the best recognition performance is combined with 8 other methods except GLCM4. Different features are fused in a cascade way. The results are shown in **Table 7**.

**Table 7:** Classification performance of GLCM1 combined with other descriptors.

Features	Magnification	Image_accuracy (%)	Patient_accuracy (%)	Sensitivity (%)	Precision (%)	F1-score (%)
GLCM1+APVEC	40X	**94.22 ± 2.18**	**93.70 ± 2.68**	95.77 ± 2.19	96.39 ± 1.57	96.07 ± 1.77
	100X	**92.78 ± 2.88**	**91.80 ± 3.67**	95.47 ± 4.45	94.83 ± 1.84	95.09 ± 2.29
	200X	94.77 ± 2.18	94.33 ± 2.99	97.23 ± 3.64	95.89 ± 2.13	96.50 ± 1.65
	400X	91.20 ± 2.17	91.59 ± 1.94	91.82 ± 3.90	96.03 ± 2.9	93.80 ± 1.80
GLCM1+ HIM	40X	**94.13 ± 1.85**	93.15 ± 2.52	95.26 ± 2.24	96.75 ± 1.33	95.99 ± 1.56
	100X	**92.69 ± 2.71**	91.17 ± 3.58	95.46 ± 4.74	94.75 ± 1.71	95.03 ± 2.22
	200X	**94.88 ± 2.39**	**94.30 ± 3.17**	96.98 ± 4.00	96.29 ± 2.47	96.56 ± 1.76
	400X	**91.19 ± 2.73**	**91.54 ± 2.26**	91.55 ± 4.75	96.26 ± 2.44	93.76 ± 2.27
GLCM1+Wavelet	40X	93.24 ± 3.45	92.85 ± 3.75	94.67 ± 3.7	96.06 ± 2.14	95.35 ± 2.61
	100X	92.37 ± 3.47	91.38 ± 4.29	95.03 ± 4.67	94.66 ± 1.98	94.80 ± 2.73
	200X	94.25 ± 1.64	93.47 ± 2.39	96.35 ± 3.45	96.06 ± 2.33	96.15 ± 1.27
	400X	90.62 ± 1.45	90.35 ± 1.52	91.49 ± 3.33	95.59 ± 3.08	93.42 ± 1.27
GLCM1+Tamura	40X	93.76 ± 2.89	93.35 ± 3.24	94.81 ± 3.14	96.64 ± 1.80	95.71 ± 2.35
	100X	92.28 ± 3.73	91.07 ± 4.44	95.63 ± 5.63	94.04 ± 1.97	94.75 ± 2.95
	200X	94.88 ± 1.89	94.45 ± 2.90	97.36 ± 2.98	95.98 ± 2.71	96.61 ± 1.30
	400X	90.97 ± 1.76	91.26 ± 1.84	92.23 ± 3.63	95.34 ± 2.91	93.69 ± 1.52
GLCM1+LBP	40X	88.02 ± 3.43	88.28 ± 3.79	90.60 ± 3.33	93.33 ± 3.70	91.83 ± 2.82
	100X	89.03 ± 4.85	89.12 ± 4.08	93.05 ± 2.69	92.30 ± 5.04	92.64 ± 3.45
	200X	89.71 ± 3.10	89.26 ± 3.22	92.49 ± 3.25	93.68 ± 2.35	93.06 ± 2.33
	400X	87.18 ± 4.14	86.42 ± 3.79	89.18 ± 3.76	93.08 ± 5.15	90.99 ± 3.15
GLCM1+CLBP	40X	93.86 ± 2.62	93.07 ± 3.55	95.69 ± 4.12	96.03 ± 2.36	95.84 ± 2.14
	100X	91.72 ± 3.07	90.46 ± 3.82	94.06 ± 3.75	94.70 ± 1.62	94.36 ± 2.39
	200X	94.34 ± 1.99	93.51 ± 2.61	97.48 ± 1.60	95.15 ± 2.21	96.29 ± 1.29
	400X	89.66 ± 3.23	88.94 ± 3.64	90.87 ± 5.22	94.83 ± 3.75	92.69 ± 2.66
GLCM1+Gabor	40X	87.63 ± 4.43	87.22 ± 4.8	93.86 ± 2.38	90.47 ± 6.57	91.92 ± 3.15
	100X	90.28 ± 2.04	89.95 ± 2.72	93.44 ± 2.97	93.71 ± 3.22	93.51 ± 1.34
	200X	91.28 ± 2.64	89.81 ± 4.09	97.06 ± 1.88	91.92 ± 3.66	94.37 ± 1.70
	400X	89.02 ± 2.83	87.80 ± 2.54	96.72 ± 1.77	89.22 ± 3.37	92.79 ± 1.96
GLCM1+Hog	40X	85.55 ± 5.78	86.56 ± 5.32	93.02 ± 2.53	88.39 ± 6.82	90.56 ± 4.20
	100X	86.62 ± 5.08	88.34 ± 3.18	94.06 ± 3.22	89.23 ± 7.32	91.35 ± 3.14
	200X	90.47 ± 3.78	90.04 ± 3.61	93.68 ± 4.40	93.55 ± 2.85	93.57 ± 2.92
	400X	88.15 ± 4.05	88.13 ± 3.72	91.45 ± 2.54	92.28 ± 4.56	91.82 ± 3.01

The bold values indicate that the recognition accuracy of combining the two features is better than that of a single feature.

**Table 7** shows that after the combination of GLCM1 and APVEC, the recognition accuracy of 40X and 100X is better than a single method whether it is for the image level or the patient level, and the accuracy of 200X and 400X is slightly lower than that of APVEC. The combination of GLCM1 and HIM improves the image level accuracy, while for the patient level, the accuracy of 40X and 100X is slightly lower than GLCM1. This shows the complementary relationship between GLCM1 and APVEC, HIM. The performance of the combination of GLCM1 and other methods is lower than that of single GLCM1, which shows that the fusion of different texture features increases the redundancy of features and reduces the recognizability.

The recognition accuracy of GLCM1, GLCM4, APVEC, and HIM based on the three-channel features is better than many existing studies, particularly, better than the performance of some deep learning models. **Table 8** shows that the method proposed in this paper is superior to many state-of-the-art methods in benign and malignant tumor recognition, both for the image level and the patient level. It is worth mentioning that works (35–43) did not split training and test set according to the protocol of (9), works (44, 45) adopted the existed protocol, and works (46, 47) randomly divided training set (70%) and test set (30%), but they did not mention whether it was the same as the protocol. Although the recognition accuracy of the works (37, 39, 41–43, 46, 47) is significantly higher than that of our method, they all use deep learning model, which requires a large number of labeled training samples and consumes longer training time. In addition, in these works, except (42), they only calculated the image level recognition accuracy. George et al. even only tested their method based on the data of 200X.

**Table 8:** Comparison of the proposed methods with other state-of-the-art methods.

Methods	Training/test	Magnification	Image_accuracy (%)	Patient_accuracy (%)
Gupta V et al. (35)	70%/30%	40X	\	87.2 ± 3.74
		100X	\	88.22 ± 3.28
		200X	\	88.89 ± 2.51
		400X	\	85.82 ± 3.81
Das K et al. (36)	80%/20%	40X	\	89.52
		100X	\	89.06
		200X	\	88.84
		400X	\	87.67
Das K et al. (37)	80%/20%	40X	94.82	\
		100X	94.38	\
		200X	94.67	\
		400X	93.49	\
Cascianelli S et al. (38)	25%/75%	40X	\	87.0
		100X	\	85.2
		200X	\	85.0
		400X	\	81.3
Wei B et al. (39)	75%/25%	40X	97.89	97.02
		100X	97.64	97.23
		200X	97.56	97.89
		400X	97.97	97.50
Zhi W et al. (40)	80%/20%	40X	91.28	\
		100X	91.45	\
		200X	88.57	\
		400X	84.58	\
Nahid AA et al. (41)	85%/15%	40X	95.0	\
		100X	96.6	\
		200X	93.5	\
		400X	94.2	\
Han Z et al. (42)	50%/50%	40X	95.8 ± 3.1	97.1 ± 1.5
		100X	96.9 ± 1.9	95.7 ± 2.8
		200X	96.7 ± 2.0	96.5 ± 2.1
		400X	94.9 ± 2.8	95.7 ± 2.2
Boumaraf S et al. (43)	80%/20%	40X	99.25	\
		100X	99.04	\
		200X	99.00	\
		400X	98.08	\
Song Y et al. (44)	70%/30%	40X	87.0 ± 2.6	90.0 ± 3.2
		100X	86.2 ± 3.7	88.9 ± 5.0
		200X	85.2 ± 2.1	86.9 ± 5.2
		400X	82.9 ± 3.7	86.3 ± 7.0
Saxena S et al. (45)	70%/30%	40X	86.41	89.46
		100X	88.92	92.61
		200X	90.05	93.92
		400X	83.16	89.78
Wang P et al. (46)	70%/30%	40X	92.71 ± 0.16	\
		100X	94.52 ± 0.11	\
		200X	94.03 ± 0.25	\
		400X	93.54 ± 0.24	\
George K et al. (47)	70%/30%	40X	\	\
		100X	\	\
		200X	96.66 ± 0.77	\
		400X	\	\

## Conclusion

In this paper, a breast cancer histopathological images recognition method based on low dimensional three-channel features is proposed. There have been many related studies, but in traditional methods, most scholars did not consider the color channel of the image, so that the extracted features lost part of the effective information. This paper compares the performance of 10 different feature descriptors in the recognition of breast cancer histopathological images. We extracted the three-channel features of different descriptors and fused the features of each channel. Then SVM was used to assess their performance. The experimental results show that the recognition accuracy of GLCM1, GLCM4, APVEC can reach more than 90% regardless of the image level or the patient level. And the performance based on three-channel features is much better than that of gray-level features, especially for GLCM1, GLCM4. We also proved that the R channel has a greater impact on the classification results of 40X, 100X, and 200X, while for 400X, it is more dependent on the B channel. In addition, high dimensional features consume more recognition time, this paper dedicates to achieving accurate recognition based on low dimensional features. Experiment results verify that the high dimensional features extracted by LBP, Hog, and Gabor require more recognition time, but the accuracy has not been greatly improved. Our method is based on the existing traditional methods and is easy to implement without complex image preprocessing. Experimental results and comparison with other methods confirm that our method requires less training time than deep learning methods, which cannot be ignored in practical applications.

In the future work, we will continue to propose more efficient and rapid methods for breast cancer recognition. The target is to realize multi-class recognition of breast cancer based on the research of benign and malignant tumor recognition. In addition to improving the recognition accuracy, we also hope to extract more effective information about cancer, which can help doctors find the lesion faster and reduce the workload on doctors.

## Data Availability Statement

Publicly available datasets were analyzed in this study. This data can be found here: https://web.inf.ufpr.br/vri/databases/breast-cancer-histopathological-database-breakhis/.

## Author Contributions

Data processing: TX and HH. Methodology: YH, YB, and SQ. Software: YH, SQ, and LZ. Supervision: YB, HH, TX, WZ and GZ. Original draft: YH. Review & editing: YH, SQ, YB, WZ and GZ. All authors contributed to the article and approved the submitted version.

## Funding

This work was supported by the National Natural Science Foundation of China as National Major Scientific Instruments Development Project (Grant No. 61927807), the National Natural Science Foundation of China (Grant No. 51875535, 61774137), the Key Research and Development Projects of Shanxi Province (Grant No. 201903D121156) and the National Key Research and Development Project (Grant No. 2019YFC0119800).

## Conflict of Interest

The authors declare that the research was conducted in the absence of any commercial or financial relationships that could be construed as a potential conflict of interest.
